# Transcranial Magnetic Stimulation of Medial Prefrontal and Cingulate Cortices Reduces Cocaine Self-Administration: A Pilot Study

**DOI:** 10.3389/fpsyt.2018.00080

**Published:** 2018-03-16

**Authors:** Diana Martinez, Nina Urban, Alex Grassetti, Dinissa Chang, Mei-Chen Hu, Abraham Zangen, Frances R. Levin, Richard Foltin, Edward V. Nunes

**Affiliations:** ^1^Columbia University College of Physicians and Surgeons, The New York State Psychiatric Institute, New York, NY, United States; ^2^Research Foundation for Mental Hygeine, New York, NY, United States; ^3^Department of Life Sciences, Ben-Gurion University of the Negev, Beer-Sheva, Israel

**Keywords:** addiction, cocaine, transcranial magnetic stimulation, prefrontal cortex, self administration

## Abstract

**Background:**

Previous studies have shown that repetitive transcranial magnetic stimulation (rTMS) to the dorsolateral prefrontal cortex may serve as a potential treatment for cocaine use disorder (CUD), which remains a public health problem that is refractory to treatment. The goal of this pilot study was to investigate the effect of rTMS on cocaine self-administration in the laboratory. In the self-administration sessions, CUD participants chose between cocaine and an alternative reinforcer (money) in order to directly measure cocaine-seeking behavior. The rTMS was delivered with the H7 coil, which provides stimulation to the medial prefrontal cortex (mPFC) and anterior cingulate cortex (ACC). These brain regions were targeted based on previous imaging studies demonstrating alterations in their activation and connectivity in CUD.

**Methods:**

Volunteers with CUD were admitted to an inpatient unit for the entire study and assigned to one of three rTMS groups: high frequency (10 Hz), low frequency (1 Hz), and sham. Six participants were included in each group and the rTMS was delivered on weekdays for 3 weeks. The cocaine self-administration sessions were performed at three time points: at baseline (pre-TMS, session 1), after 4 days of rTMS (session 2), and after 13 days of rTMS (session 3). During each self-administration session, the outcome measure was the number of choices for cocaine.

**Results:**

The results showed a significant group by time effect (*p* = 0.02), where the choices for cocaine decreased between sessions 2 and 3 in the high frequency group. There was no effect of rTMS on cocaine self-administration in the low frequency or sham groups.

**Conclusion:**

Taken in the context of the existing literature, these results contribute to the data showing that high frequency rTMS to the prefrontal cortex may serve as a potential treatment for CUD.

## Introduction

Brain stimulation techniques, such as repetitive transcranial magnetic stimulation (rTMS), have been proposed as potential therapies for cocaine use disorders (CUD) (1). Previous studies have shown that rTMS to the dorsolateral prefrontal cortex (dlPFC) reduces craving for cocaine (2–4) and recent preliminary clinical trials have indicated that rTMS to the dlPFC may improve abstinence from cocaine (5, 6).

In this study, we investigated the effect of rTMS on cocaine-seeking behavior using methods that differed from previous research, but provide complementary findings. First, we used cocaine self-administration sessions as the outcome measure, where participants choose between smoked cocaine and an alternative reinforce (money) in the laboratory. This type of study provides behavioral data on the safety and efficacy of potential treatments in a small number of volunteers under controlled laboratory conditions (7). Second, we delivered rTMS with the H7 coil (Brainsway, Inc., Jerusalem, Israel), which has not been used in previous studies of CUD. Most previous rTMS studies in CUD have used figure 8 coils that provide focal stimulation to discrete brain regions, such as the dlPFC (2–6). The H coil induces a magnetic field with a deeper and wider distribution than other types of coils (8, 9). We specifically used the H7 coil to target the medial prefrontal cortex (mPFC) and the dorsal anterior cingulate (dACC) (10). This coil was used to allow stimulation of these broad brain regions, given that previous studies have shown that, while the dlPFC is involved in CUD, the mPFC and dACC are crucial for regulating cognitive control over decisions, particularly when those decisions require overcoming pre-potent or habitual tendencies (11, 12).

Previous imaging studies have shown that CUD is associated with alterations in activation or connectivity in the dorsal attention, executive control, and salience networks, which include the dlPFC, mPFC, and dACC. Brewer et al. (13) showed an inverse relationship between the dlPFC activation and treatment retention using a Stroop task and fMRI. A more recent study demonstrated that functional connectivity between right temporal pole and mPFC-predicted relapse in CUD subjects, suggesting that deficits in the salience network are associated with impaired processing of physiologically salient events (14). Using an inhibitory control task, Golstein et al. (15) showed that hypoactivation of the dACC was associated with more frequent cocaine use (16). Imaging studies incorporating TMS have shown that stimulation of the mPFC with TMS in CUD subjects resulted in less activation in the limbic system, compared to controls, suggesting that this network is slow to engage (12, 17). Overall, these imaging studies suggest that these regions of the prefrontal cortex, the dlPFC, mPFC, and dACC, play a role in decision making, cue response, and cognitive control CUD. Thus, an advantage of the H coil is the ability to simultaneously stimulate these brain regions.

In this pilot study, volunteers with CUD were admitted to an inpatient unit and participated in cocaine self-administration sessions before, during, and after 3 weeks of rTMS. In the cocaine self-administration sessions, participants were asked to choose between smoking a low dose of cocaine (12 mg) or receiving money ($5), in order to measure cocaine-seeking behavior. The volunteers were randomized to one of three groups: high frequency (10 Hz), low frequency (1 Hz), or sham stimulation. Our hypothesis was that active high frequency rTMS would reduce cocaine self-administration compared to the sham condition. In addition, given the differential effect on neural excitability of high versus low frequency stimulation (18), we investigated whether the frequency of stimulation (high versus low) would affect the choice to self-administer cocaine.

## Materials and Methods

The study was approved by the Institutional Review Board of the New York State Psychiatric Institute and all participants provided written informed consent. Volunteers with cocaine use disorder (CUD), moderate to severe per DSM-5 criteria, were enrolled in the study. All participants were medically healthy and had no other current Axis I diagnosis, including abuse or dependence to other substances with the exception of caffeine and/or nicotine. The CUD subjects were actively using smoked cocaine at study entry (verified by urine toxicology) and could not be seeking treatment for their cocaine use. All subjects were admitted to an inpatient unit for the entire study (about 3 weeks).

Following admission, participants underwent 3–5 days of initial monitored abstinence, followed by the self-administration sessions and rTMS. The procedures included: (1) baseline cocaine self-administration sessions (session 1); (2) a second cocaine self-administration session performed after 4 days (during week 1) of rTMS (session 2); (3) a third cocaine self-administration session performed at the end of 13 rTMS sessions (the end of week 3, session 3). The rTMS was delivered on weekdays, over the course of 3 weeks. Due to safety concerns regarding the potential interaction between rTMS and cocaine, rTMS was not delivered on the same days as the cocaine self-administration sessions.

### Cocaine Self-Administration Sessions

**Figure 1:**
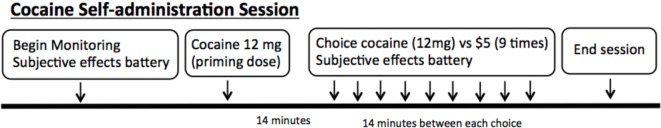
Schematic of the cocaine self-administration sessions in the laboratory. Each session begins with monitoring and subjective effects battery. A “priming dose” of cocaine is delivered, followed by nine options for the subjects to choose between 12 mg smoked cocaine or $5.

The self-administration sessions were performed in our research laboratory as established previously by our group (19–22). A schematic of the session is shown in Figure 1. For safety purposes, all self-administration sessions included repeated ECG, recording of vital signs, and monitoring *via* a one-way mirror by a physician. All sessions were performed with 12 mg doses of smoked cocaine. Cocaine hydrochloride was purchased from Mallinckrodt and prepared into cocaine base by our research pharmacy.

The sessions began with each volunteer receiving an experimenter delivered free (no response requirement) or “priming” dose of smoked cocaine (12 mg). This was provided prior to making choices, so that the participants had sampled the dose of cocaine that would be available for the day’s session. Following the priming dose, the volunteers were presented with the choice between smoked cocaine (12 mg) and money ($5) nine times during each session. In addition, subjects were required to press the space bar on a computer keyboard in order to receive their choice. The number of presses required to obtain the dose increased using a progressive ratio schedule (e.g., 200, 400, 600, 800) in order to reflect a subject’s motivation to obtain their choice over the series of choices. The outcome measure was the number of doses chosen and taken during each session. Participants were asked to rate their craving for cocaine prior to each session with a visual analog scales labeled “not at all” at 0 mm and “extremely” at 100 mm, as previously described (23).

### Transcranial Magnetic Stimulation

The H7 coil was provided by Brainsway, Inc., (Jerusalem, Israel) along with the Magtsim Rapid stimulator (Morrisville, NC, USA). All sessions began with ascertainment of the resting motor threshold (rMT), obtained by applying TMS pulses over the motor cortex (leg motor area) to induce activation of the tibialis muscle of the lower extremity. The rMT was the lowest stimulus intensity that elicited a response in the leg, measured either by motor evoked potential or visible movement of the leg. The rMT was obtained each day that rTMS was delivered. Once the position of the rMT was determined, the positioning for the prefrontal cortex was obtained by moving the coil anteriorly from the point where the MT was obtained, as described previously (10).

High frequency stimulation was delivered at 10 Hz with a train duration of 3 s (for 40 trains per session), an inter-train interval of 20 s, and a total of 1,200 pulses per session. Low frequency was delivered using a standard 1 Hz protocol that includes 900 pulses per session. For the sham condition, a sham coil is present within the same TMS helmet along with the active magnetic coil. The sham coil produces a similar clicking noise and delivers superficial stimulation to the scalp intended to mimic sensations produced by active high frequency rTMS. The subjects performed no cue or cognitive task during the rTMS. The fidelity of the sham condition was not evaluated with a questionnaire. However, none of the subject were familiar with TMS and none reported that they suspected sham.

Initially, the active rTMS was delivered at 100% of motor threshold on day 1, then increased to 110% on day 2, and to 120% at day 3. However, it was quickly determined that many of the CUD participants could not tolerate the rTMS at greater than 100% of MT delivered at high frequency. Four subjects dropped out in the first 3 days of rTMS delivery due to the aversive sensation associated with increasing to 120% of MT. Thus, the protocol was modified to begin at 90% of MT with an increase to 110% over 2–3 days. Additionally, the determination of motor threshold was separated in time from the rTMS session: the determination of MT was done in the morning and the rTMS sessions in the afternoon. Since determining the MT requires stimulation, separating this from the rTMS session reduced the number of stimuli delivered within the same time period. With this modification, participants reported far less discomfort and were able to complete the study.

### Data Analysis

The primary outcome variable was the count of the number of doses of cocaine chosen during each cocaine self-administration session, with a minimum of 0, and maximum of 9 possible choices. The second outcome was craving scores ranged 0–100. Because the outcome variables had non-normal distribution, a negative binomial distribution with random effect for each subject was modeled as a function of baseline score (number of cocaine doses chosen or craving score during session 1), treatment condition (between subjects: sham, low frequency, high frequency), and occasion within subjects: session 2 (after 4 days of rTMS) and session 3 (after 13 days of rTMS). The interaction between treatment and occasion was tested and remained in the final models if significant at *p* < 0.05. SAS 9.4 was used in all analyses.

## Results

A total of 18 volunteers completed the study, with 6 in the high frequency group, 6 in the low frequency group, and 6 in the sham group. The demographics of each group are shown in Table 1.

**Table 1:** Demographic data of the three repetitive transcranial magnetic stimulation groups of subjects with cocaine use disorder (*N* = 18).

	HF	LF	Sham
Age (in years): mean (SD)	42 (7)	44 (5)	44 (6)
Gender (male/female): *n*	6 M	6 M	5M/1F
Ethnicity: *n*	4AA/1C/1H	6AA	6AA
Years of cocaine use: mean (SD)	19 (9)	21 (9)	20 (9)
Weekly spending on cocaine (US$): mean (SD)	292 (157)	388 (305)	210 (102)

*HF, high frequency; LF, low frequency; M, male; F, female; AA, African–American; C, Caucasian; H, Hispanic*.

The results from the self-administration sessions are reported in Table 2. The mixed effects model on the outcome of number of cocaine doses chosen during cocaine self-administration sessions yielded a significant interaction of treatment by occasion [*F*(2, 15) = 5.36, *p* = 0.02]. As can be seen in the Table 2, there was little change in cocaine self-administration in the sham group or in the low frequency group across the three sessions. There was a tendency for the cocaine choices to increase slightly from baseline to session 2 (after 4 days of treatment) in each group. In the high frequency group there was also a slight increase between session 1 and session 2, but the number of choices drops at session 3.

**Table 2:** The number of choices participants made for smoked cocaine by three repetitive transcranial magnetic stimulation (rTMS) groups (Sham, low frequency, or high frequency treatment) on cocaine self-administration sessions.

	High frequency	Low frequency	Sham

	Mean (SD)		
**Choices for cocaine**			
Session 1	3.83 (4.0)	4.7 (3.3)	4.8 (3.8)
Session 2	5.0 (2.5)	6.5 (2.7)	5.0 (3.9)
Session 3	1.8 (1.9)*	5.5 (3.5)	4.7 (4.0)

**Break point**			
Session 1	1,333.33 (1,608.31)	1,700 (1,275.93)	1,766.67 (1,477.39)
Session 2	1,800 (1,011.93)	2,400 (1,095.45)	1,833.33 (1,488.17)
Session 3	600 (704.27)	2,033.33 (1,341.14)	1,733.33 (1,521.40)

*Session 1 is the baseline (pre-rTMS session), session 2 followed 4 days of rTMS, and session 3 occurred following 13 days of rTMS. Treatment by session interaction with rTMS group is significant [*F*(2, 15) = 5.36, *p* = 0.02]*.

*Session 2 compared to session 3, p = 0.03.

Within the high frequency group, the choice for cocaine was lower at session 3 compared to session 2 (*t* = 4.00, *p* = 0.001). The difference in choices for cocaine between high frequency and low frequency groups, at session 3, was significant (*t* = 2.31, *p* = 0.04), while difference between the high frequency and sham condition, at session 3, approached significance (*t* = 1.84, *p* = 0.09).

The break point from the progressive ratio is included in Table 2. Within the high frequency condition break point was lower at session 3 compared to session 2 (*t* = 4.04, *p* = 0.001). Differences in break point between high frequency and low frequency at session 3 were significant (*t* = 2.37, *p* = 0.03), while differences between high frequency and sham conditions approaches significance (*t* = 1.94, *p* = 0.07).

The rMT was obtained one each day that rTMS was delivered. The average rMT obtained per subject is provided in Table 3, and is reported as the percent output of the Magstim Rapid Stimulator. The rMT did not change significantly over time [*F*(12, 194) = 0.74, *p* = 0.71].

**Table 3:** Resting motor threshold (RMT) per subject.

High frequency	RMT average (SD), %
HF1	71.1 (2.9)
HF2	81.6 (3.0)
HF3	66 (4.2)
HF4	92.9 (2.1)
HF5	63.8 (3.3)
HF6	86.6 (5.3)

**Low Frequency**	
HF1	84.3 (4.2)
HF2	78 (6.3)
HF3	87.1 (4.9)
HF4	74.8 (4.0)
HF5	89.3 (2.4)
HF6	72.3 (3.2)

**Sham**	
S1	73.6 (3.5)
S2	82.0 (2.5)
S3	95 (0.8)
S4	68 (2.9)
S5	88 (2.6)
S6	64.2 (2.1)

*The RMT was obtained each day that repetitive transcranial magnetic stimulation was delivered and is reported as the percent output of the Magstim Rapid stimulator machine (Morrisville, NC, USA). RMT did change significantly over time [*F*(12, 194) = 0.74, *p* = 0.71]*.

Craving was not affected by any rTMS condition. Baseline measures of craving varied between the three groups at baseline (prior to rTMS). The high frequency group reported greater ratings of craving (Mean = 61.0, SD = 38.1), compared to the low frequency group (Mean = 21.7, SD = 28.8) and the sham group (Mean = 16.8, SD = 25.0) [*F*(2,15) = 6.29, *p* = 0.01]. For all three rTMS conditions, the craving scores at session 3 were lower than session 2 [*F*(1, 17) = 12.08, *p* = 0.003]. However, rTMS did not affect subsequent measures of craving [*F*(2, 14) = 0.77, *p* = 0.48].

## Discussion

In this pilot study, we demonstrated that delivering high frequency rTMS over a 3-week period, using an H7 coil targeting the mPFC and dACC, is feasible and safe in volunteers with CUD. Further, the data are suggestive of a beneficial effect of high frequency rTMS in reducing the choice for smoked cocaine after 3 weeks of treatment, compared to low frequency rTMS or sham. Both low frequency and sham resulted in little change in the number of choices for cocaine across the three study sessions. The significant session by treatment interaction was carried by the large reduction in choice for cocaine the high frequency treatment group between 1 week and 3 weeks.

Previous studies have shown that rTMS stimulation of the dlPFC reduces craving for cocaine (2–4). One study reported that two sessions of high frequency rTMS applied to the right dlPFC reduced craving, although the effect dissipated after 4 h (2). Another study showed that high frequency rTMS, applied to the left dlPFC, had no acute affect on craving, but reduced it gradually over 10 daily sessions (3). The third study used high frequency rTMS, with the H1 coil directed bilaterally at the dlPFC (with preference to the left hemisphere), as an add-on treatment in the clinic for CUD, and showed that craving was reduced gradually over a month of treatment (4). We measured craving, but saw no effect of rTMS or sham. However, there were large between group differences at baseline, prior to rTMS, which make the data hard to interpret.

Recent small-scale clinical trials have indicated that high frequency rTMS to the prefrontal cortex may improve abstinence from cocaine. Terraneo et al. (6) enrolled 32 CUD subjects who were randomized to receive either high frequency rTMS (15 Hz) to the dlPFC with the figure 8 coil or the control condition, consisting of pharmacological treatment. The results showed that the rTMS group had more cocaine-free urine drug tests compared to the control condition. Similar results were published by Bolloni et al. (5), who performed a study in CUD subjects treated with 1 month of high frequency (10 Hz) rTMS to the bilateral dlPFC, using the H1 coil, compared to sham. The results showed a reduction on cocaine use, measured with hair analysis, in the rTMS group at 3–6 months, although no difference was seen between the groups at one month, which was immediately following treatment.

The results of our study compliment these findings in two ways. The first is that we used cocaine self-administration sessions in the laboratory, which provides a direct measure of each participants’ choice for cocaine when presented with an alternative reinforcer. This method has not been used previously to investigate the effect of rTMS on cocaine self-administration. The second is that we used the H7 coil, which distributes stimulation to deeper and wider regions of the prefrontal cortex compared to figure 8 coils (8, 9). We used the H7 coil to stimulate mPFC and dACC, based on research showing that these regions play a significant role in CUD.

In this study, we found it necessary to modify our initial protocol for high frequency rTMS delivered with the H7 coil. Participants had difficulty tolerating stimulation that increased from 100 to 120% of MT, and we amended the protocol to address this issue by lowering the maximal stimulation, as described in the methods. One reason for the difficulty tolerating the higher stimulation may be that cocaine use is associated with a higher MT compared to controls, requiring higher amperage output from the stimulator, as has been reported in previous studies (24–26). In this study, we did not use a questionnaire to evaluate the subjects expectation of sham versus active treatment, although none of the subjects were familiar with TMS. This study also did not include a cognitive task with the delivery of rTMS.

Overall, these results show that rTMS using the H7 coil is feasible, and shows preliminary efficacy in decreasing cocaine-seeking behavior. These data also indicate that low frequency stimulation with the H7 coil did not have an impact on cocaine self-administration. The results from this pilot study indicate that HF rTMS with the H7 coil directed at the mPFC and dACC may reduce the choice to take cocaine. These results, combined with previous studies, suggest that rTMS may provide a viable treatment option for this disorder, for which there are few options. Future studies are warranted, with larger samples sizes, including female subjects, which this study did not include.

## Ethics Statement

The study was approved by the Institutional Review Board of the New York State Psychiatric Institute and all participants provided written informed consent.

## Author Contributions

DM served as the principal investigator for the study. NU provided clinical coverage of subjects. AG and DC screened subjects and performed the rTMS. M-CH provided the statistical analysis. AZ designed the rTMS portion of the study. FL oversaw the clinical management of subjects and manuscript preparation. RF designed and oversaw the cocaine self-administration portion of the study. EN assisted DM in the overall management of the study.

## Conflict of Interest Statement

AZ is a co-inventor of the TMS H7 coil and serves as consultant for, and has financial interests in, Brainsway. The other authors report no competing financial interest in relation to the study design, results, or discussion.
